# The diagnostic utility of IL-10, IL-17, and PCT in patients with sepsis infection

**DOI:** 10.3389/fpubh.2022.923457

**Published:** 2022-07-22

**Authors:** Wei Zhang, Weiwei Wang, Weiwei Hou, Chenfei Jiang, Jingwen Hu, Li Sun, Liqing Hu, Jian Wu, Anquan Shang

**Affiliations:** ^1^Department of Laboratory Medicine, Shanghai Tongji Hospital, School of Medicine, Tongji University, Shanghai, China; ^2^Department of Laboratory Medicine, Jiaozuo Fifth People's Hospital, Jiaozuo, China; ^3^Department of Laboratory Medicine, Tinghu People's Hospital of Yancheng City, Yancheng, China; ^4^The College of Medical Technology, Shanghai University of Medicine and Health Sciences, Shanghai, China; ^5^Department of Medical Laboratory Technology, School of Medicine, Xiangyang Polytechnic, Xiangyang, China; ^6^Department of Laboratory Medicine, Ningbo First Hospital and Ningbo Hospital, Ningbo, China; ^7^Department of Laboratory Medicine, The Affiliated Suzhou Hospital of Nanjing Medical University, Suzhou Municipal Hospital, Gusu School, Nanjing Medical University, Suzhou, China

**Keywords:** sepsis, diagnostic value, IL-10, IL-17, PCT, nomogram

## Abstract

**Objective:**

The purpose of this study is to determine the diagnostic value and net clinical benefit of interleukin-10 (IL-10), interleukin-17 (IL-17), procalcitonin (PCT), and combination tests in patients with sepsis, which will serve as a standard for sepsis early detection.

**Patients and methods:**

An investigation of 84 sepsis patients and 81 patients with local inflammatory diseases admitted to the ICU of Tongji University Hospital in 2021. In addition to comparing inter-group variability, indicators relevant to sepsis diagnosis and therapy were screened.

**Results:**

LASSO regression was used to examine PCT, WBC, CRP, IL-10, IFN-, IL-12, and IL-17. Multivariate logistic regression linked IL-10, IL-17, and PCT to sepsis risk. The AUC values of IL-10, IL-17, PCT, and the combination of the three tests were much higher than those of standard laboratory infection indicators. The combined AUC was greater than the sum of IL-10, IL-17, and PCT (*P* < 0.05). A clinical decision curve analysis of IL-10, IL-17, PCT, and the three combined tests found that the three combined tests outperformed the individual tests in terms of total clinical benefit rate. To predict the risk of sepsis using IL-10, IL-17, and PCT had an AUC of 0.951, and the model's predicted probability was well matched. An examination of the nomogram model's clinical value demonstrated a considerable net therapeutic benefit between 3 and 87%.

**Conclusion:**

The IL-10, IL-17, and PCT tests all have a high diagnostic value for patients with sepsis, and the combination of the three tests outperforms the individual tests in terms of diagnostic performance, while the combined tests have a higher overall clinical benefit rate.

## Introduction

Sepsis is described as a potentially fatal organ malfunction produced by an abnormally activated host response to infection (1). In sepsis, the body's immunological response to an invading pathogen fails to restore to equilibrium, leading to a pathological condition marked by long-term inflammation and immune suppression (2). As a potentially life-threatening condition with a high global frequency (3), sepsis was diagnosed in almost 50 million people globally in 2017, with an estimated 11 million sepsis-related fatalities (4). The mortality rate of severe sepsis is around 30–50%, and Septic shock has a mortality rate of more than 50% (5). Therefore, early and accurate diagnosis of sepsis plays an important role in successful treatment and improving survival, however, the signs and symptoms of sepsis are similar to non-infectious inflammatory reactions, which brings greater difficulties in clinical diagnosis when the source of infection cannot be identified. For diagnosing a bacterial illness, microbial cultures are the “gold standard” (6), Due to the fact that sepsis is frequently caused by infection, the diagnosis of sepsis can be confirmed by performing a blood culture to identify the pathogenic bacteria. However, reports show that the positive blood culture rate is only about 10% in infectious diseases and 30–40% in patients with sepsis (7–9). The Journal of the American Medical Association published the third international consensus on the definition of sepsis and septic shock in 2016 (10), defining sepsis as life-threatening organ dysfunction caused by a dysregulated host response to infection, with organ dysfunction defined as an acute change in sequential organ failure assessment (SOFA) ≥2 caused by infection, i.e., Sepsis = Infection + SOFA ≥ 2. Although the diagnostic criteria for sepsis are clearly established, it has been argued that the SOFA score has a lag and that most patients are already in an advanced state of sepsis when identified with the SOFA score (11). As a result, it is yet unknown how to develop a simple diagnostic index with high diagnostic effectiveness in the early stages of sepsis.

Interleukin 10 (IL-10) is a pleomorphic cytokine with numerous phenotypic roles that is released by granulocytes, dendritic cells, macrophages, B cells, and T cell subsets (12). IL-10 is a major anti-inflammatory cytokine with immunomodulatory effects that suppresses pro-inflammatory cytokine production (13) and has a role in the pathophysiology of autoimmune disorders (14, 15). Interleukin 17 (IL-17) is a T-cell-derived cytokine that is generated by macrophages, dendritic cells, mast cells, and natural killer cells (16). IL-17 is a pro-inflammatory cytokine that binds to corresponding receptors, leading to inflammatory cell infiltration and tissue damage (17), IL-17 is involved in host defense against infection as well as the development of inflammatory disorders (18).

Serum procalcitonin (PCT), a precalcitonin peptide body, is raised during inflammation and infection and is used as an early infection diagnostic test (19). One of the established processes of sepsis is an imbalance in the body's anti-inflammatory defenses, and the early phases of sepsis increase inflammatory factor release. To find out if IL-10, IL-17, PCT, and combined tests have a diagnostic value, this study will use the third international consensus criteria as diagnostic criteria (10). It will also find out what value an optimal infection level is for sepsis patients and evaluate whether IL-10, IL-17, and PCT have a net clinical benefit in patients with sepsis infection. Finally, a column line chart will be developed to predict the risk of sepsis using data from these tests.

## Materials and methods

### Patients and clinical information

The sepsis group was established using a retrospective review of 84 patients with sepsis admitted to the ICU at Tongji Hospital, School of Medicine, Tongji University in 2021. Sepsis was diagnosed using the third international consensus diagnostic criteria for sepsis and infectious shock 3.0, which was published in 2016. The infection group included 81 inpatients hospitalized within the same time with non-sepsis and local inflammatory infection. The infection group had two or more of the following symptoms: body temperature >38°C or 36 < °C; heart rate >90 beats/min; respiratory rate >20 breaths/min or arterial blood carbon dioxide partial pressure <32 mmHg; peripheral blood leukocytes >12 × 10^9^/L or <4 × 10^9^/L. Sepsis was defined as having both the fundamental indications of infection and a SOFA score ≥2.

The exclusion criteria were as follows: Oncology patients, hematology patients, organ transplant patients, patients with incomplete clinical and laboratory data, and patients with immunological weakness are excluded from the study.

### Clinical information and laboratory examination

Clinical and laboratory data of included patients, including age, gender, PCT, white blood cells (WBC), C-reactive protein (CRP), interleukin-1β (IL-1β), interleukin-2 (IL-2), interleukin-4 (IL-4), interleukin-5 (IL-5), interleukin-6 (IL-6), interleukin-8 (IL-8), interleukin-10 (IL-10), interleukin-12 (IL-12), interleukin-17 (IL-17), tumor necrosis factor-a (TNF-α), interferon-a (IFN-α), interferon gamma (IFN-γ), and SOFA score. The tests included were based on the initial serum sample taken within 24 hours of the patients' admission to the clinic. PCT was detected using the Roche E411 assay. WBC and CRP were detected with the Myriad BC7500. The expression levels of IL-1L, IL-2, IL-4, IL-5, IL-6, IL-8, IL-10, IL-12, IL-17, TNF-α, IFN-α, and IFN-γ were measured by Cytek^®^NL-CLC using multiplex microsphere flow immunofluorescence and double antibody sandwich assay. Antibodies are matched to the instrument (Cytek Biosciences, Inc.). Because the data were evaluated retroactively, the participants' written informed consent was waived.

### Statistical analysis

SPSS 25.0 was used to statistically evaluate the data that matched the criteria, and the normality test was utilized to assess the measurement data based on the Shapiro-Wilk test. Data with a normally distributed distribution were reported as ($\bar{x}$ ± SD), using two independent samples. The *t*-test was used to compare groups of regularly distributed measuring data, while the 2-test was used to compare counting data. The skewed distribution of the measurement data was represented by the median (M) and percentile (P25, P75), and the Mann-Whitney U test was used to compare groups of the skewed distribution of the measurement data. Spearman's rank correlation analysis was used to examine the relationships between IL-10, IL-17, PCT levels, laboratory-related markers, and SOFA scores. The sensitivity, specificity, best critical value, Youden index, negative predictive value (NPV), and positive predictive value (PPV) of each correlation index in patients with sepsis infection were determined using Graphpad prism software, and the accuracy of the test was judged by the area under the curve (AUC). The joint predictors of IL-10, IL-17, and PCT were calculated using binary logistic regression analysis, and the AUC of each index was compared using the Delong test. To assess the net clinical benefit of IL-10, IL-17, PCT, and the three combined trials, the clinical decision curve analysis (DCA) was utilized. The net clinical benefit rate of the model was estimated by subtracting the percentage of false-positive patients from the proportion of true-positive patients and comparing the relative harms of foregoing the intervention against the negative consequences of unneeded intervention. Least absolute shrinkage and selection operator (LASSO) regression, Harrell concordance index (C-index), Hosmer-Lemeshow test, Calibration calibration curve, and DCA analysis were performed on the data using R software 4.1.0 (https://www.r-project.org/). The concordance index (C-index), Hosmer-Lemeshow test, Calibration calibration curve, DCA analysis, LASSO regression screening and downscaling of age, gender, and laboratory examination indexes, screening out variables with non-zero regression coefficients, and using the 10-fold cross-validation method to select the optimal Lambda parameters Significant variables identified by LASSO regression analysis were submitted to multiple covariance analysis, and the correlates that did not have multiple covariance were included in multi-factor logistic regression analysis to identify independent influencing factors for the development of sepsis. Column plots based on IL-10, IL-17, and PCT were created to predict the risk of sepsis, and the model was tested for discrimination and calibration, with discrimination assessed by calculating the AUC values under the ROC curve. The calibration degree of the prediction model refers to the consistency between the predicted probability and the actual observed value, and it is analyzed by the Calibration calibration curve as well as the calculation of the C-index and Hosmer-Lemeshow test results to analyze the consistency between the risk prediction and the actual observed value. The closer to one the prediction model's discriminatory ability and consistency. The glmnet, rms, foreign, rmda, and survivor packages in R software 4.1.0 were utilized for validation using the Bootstrap approach with 1,000 internal duplicate samples in this work. *P* < 0.05, differences were judged statistically significant.

## Results

### Clinical baseline information was compared between patients in the infection and sepsis groups

Supplementary Figure S1 is the screening flow chart of patients in the infection group and the sepsis group. Gender, age, IL-4, IL-5, TNF-α, and IFN-α levels were not significantly different between the two groups (P > 0.05). Patients in the sepsis group had higher PCT, WBC, CRP, IL-1β, IL-2, IL-6, IL-8, IL-10, IL-12, IL-17, IFN-γ, and SOFA scores than those in the infection group, and the difference was statistically significant (*P* < 0.05) (Table 1).

**Table 1:** Comparison of the distribution of clinical data between the infection group and the sepsis group.

**Items**		**Infection group (*****n** =* **81)**	**Sepsis group (*****n** =* **84)**	χ^2^**/z value**	***P*** **-value**
Gender	Male	39	47	1.006	0.316
	Female	42	37		
Age (year)		64 (55, 74)	71 (54, 79)	0.835	0.488
WBC (×109/L)		6.64 (5.34, 8.82)	11.61 (7.87, 17.40)	3.083	<0.001
PCT (ng/mL)		0.45 (0.32, 0.87)	4.95 (2.06, 23.11)	4.567	<0.001
CRP (10mg/L)		40.20 (8.11, 64.20)	90.35 (45.90, 159.89)	2.964	<0.001
IL-1β (pg/mL)		0.56 (0.54, 0.82)	0.81 (0.54, 3.03)	2.356	<0.001
IL-2 (pg/mL)		1.01 (0.46, 2.18)	2.28 (0.64, 43.14)	2.039	<0.001
IL-4 (pg/mL)		1.10 (0.97, 1.27)	1.13 (0.98, 1.38)	0.881	0.420
IL-5 (pg/mL)		1.58 (1.19, 1.58)	1.58 (1.19, 1.58)	0.549	0.924
IL-6 (pg/mL)		1.18 (0.68, 13.15)	34.17 (3.32, 102.08)	2.775	<0.001
IL-8 (pg/mL)		1.06 (0.94, 3.29)	13.25 (2.61, 43.18)	0.304	<0.001
IL-10 (pg/mL)		0.84 (0.37, 1.38)	3.60 (1.42, 7.50)	3.573	<0.001
IL-12 (pg/mL)		1.31 (1.17, 1.46)	1.41 (1.28, 1.68)	1.489	0.024
IL-17 (pg/mL)		1.32 (1.19, 1.52)	2.66 (1.71, 5.15)	4.018	<0.001
TNF-a (pg/mL)		1.24 (1.24, 1.24)	1.24 (1.24, 1.24)	0.535	0.937
IFN-a (pg/mL)		1.13 (0.36, 1.13)	1.13 (0.92, 1.30)	0.957	0.319
IFN-γ (pg/mL)		2.18 (1.57, 4.15)	4.52 (2.30, 10.00)	2.104	<0.001
SOFA score (point)		0 (0, 0)	5 (4, 7)	6.422	<0.001
PaO2/FiO2 (mmHg)		453 (427, 469)	368 (288, 415)	4.502	<0.001
GCS score		15 (15, 15)	14 (13, 15)	3.287	<0.001
Arterial pressure (mmHg)		93 (87, 99)	73 (65, 87)	3.873	<0.001
TBIL (umol/L)		12.1 (8.6, 15.4)	24.85 (13.35, 42.25)	3.737	<0.001
PLT (×109/L)		212 (179, 248)	132 (96, 160)	4.018	<0.001
Cre (umol/L)		82 (72, 98)	133 (93, 208)	3.398	<0.001
**Underlying disease**					
Diabetes		28	31	0.098	0.754
Hypertension		32	35	0.080	0.778
Respiratory disease		10	11	0.021	0.885
Cardio-cerebrovascular diseases		12	18	1.213	0.271
Null		33	30	0.441	0.506
**Infection site**					
Respiratory system		37	40	0.062	0.803
Urinary system		25	30	0.437	0.509
Cavity		12	15	1.646	0.199
Soft tissue		8	10	0.006	0.939
Central nervous system		4	5	–	0.717
**Admitting diagnosis**					
Pulmonary infection		38	39	0.004	0.950
Urinary infection		19	24	0.560	0.454
Abdominal infection		19	17	0.250	0.617
Bloodstream infection		5	4	–	0.743
Mechanical ventilation		0	7	–	0.014
APACHE-II		10 (8, 14)	17 (8, 20)	7.640	<0.001

### Screening of independent variables based on lasso regression

**Figure 1:**
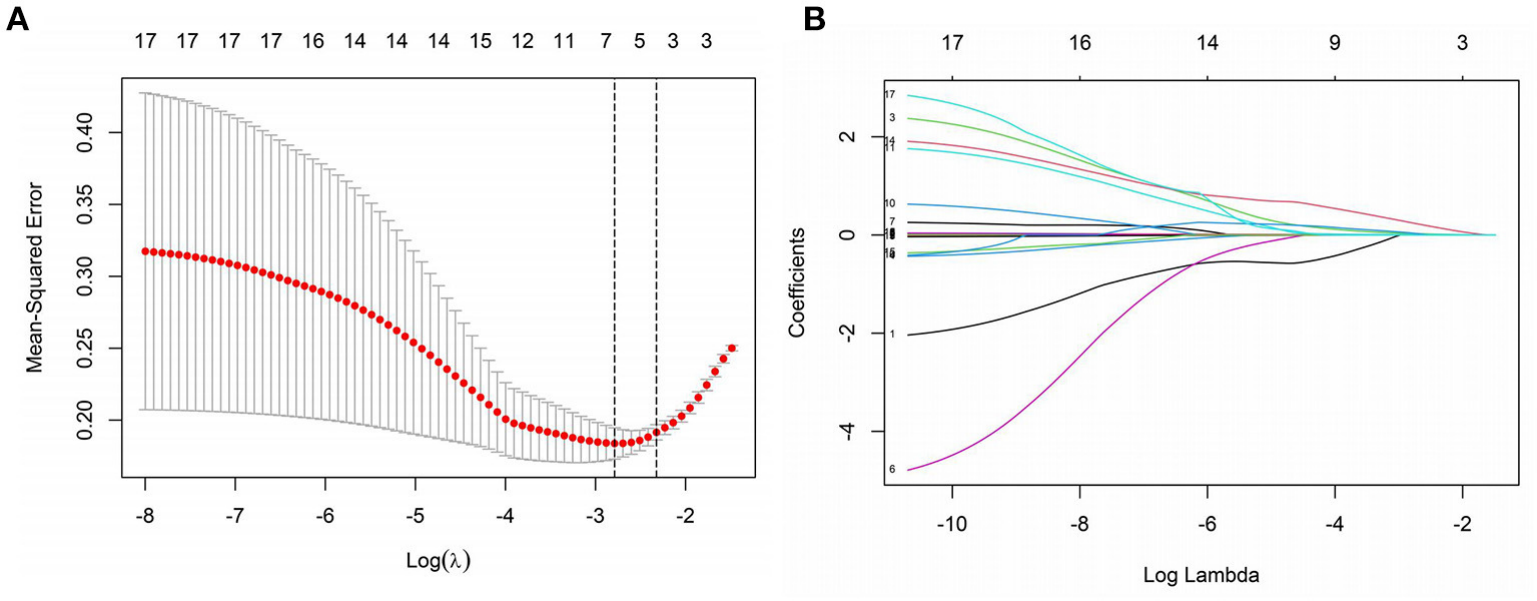
Screening of independent variables based on LASSO regression. **(A)** The optimal value of parameter λ is screened in the LASSO model using the minimum criterion for 10-fold cross-validation [the two dashed lines in the figure indicate the λ and 1-se values of the minimized mean square error (MSE)]. **(B)** LASSO regression screening variables profile (Lambda optimal take when screening retention variables).

The research examined 17 infection-related factors, including age, gender, and laboratory testing. LASSO regression was used to screen and downscale the 17 independent variables to select the characteristic variables of sepsis occurrence due to the large number of study variables to be screened included in this study, the correlation between different variables, and the limited sample size of two groups of patients to avoid multicollinearity between redundant variables. Because SOFA score was used as the grouping criterion for the response variable, i.e., the dependent variable was a dummy variable transformed from SOFA, and the two variables were non-independent states, they were not included in the independent variable screening. The 10-fold cross-validation method was used to select the optimal Lambda parameter, and the best results of the screened variables were obtained when Lambda was set to optimal 0.06752097, at which time seven variables with non-zero regression coefficients were screened, namely PCT, WBC, CRP, IL-10, IFN-γ, IL-12 and IL-17 (Figure 1).

### Multifactorial logistic regression analysis of the risk of sepsis in infected patients

**Figure 2:**
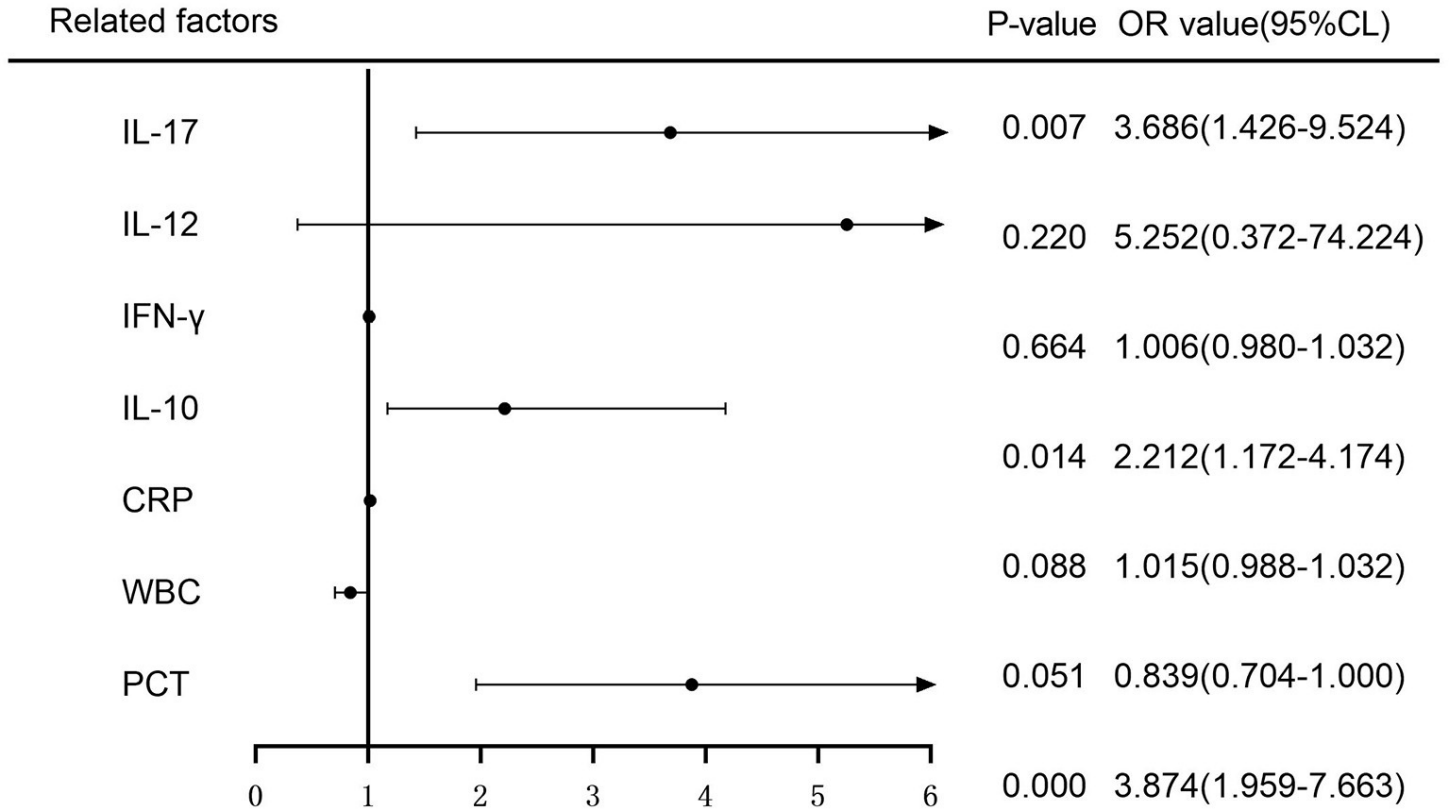
Multifactorial logistic regression analysis of the risk of sepsis development forest plot.

The variance inflation factors (VIFs) for the seven variables (PCT, WBC, CRP, IL-10, IFN-, IL-12, and IL-17) assessed by LASSO regression analysis were 1.658, 1.916, 1.374, 1.594, 1.076, 1.053, and 1.092, demonstrating the lack of multicollinearity. On these seven factors, a multifactorial logistic regression analysis was undertaken, with the incidence of sepsis as the response variable (0 = no, 1 = yes) and the seven variables screened by the LASSO regression analysis as independent variables. The multifactorial logistic regression analysis forest plot revealed that PCT, IL-10, and IL-17 were independent risk factors for the development of sepsis (*P* < 0.05) (Figure 2).

### Correlation of IL-10, IL-17 and PCT levels with other laboratory-related infection indicators in two groups of patients

**Figure 3:**
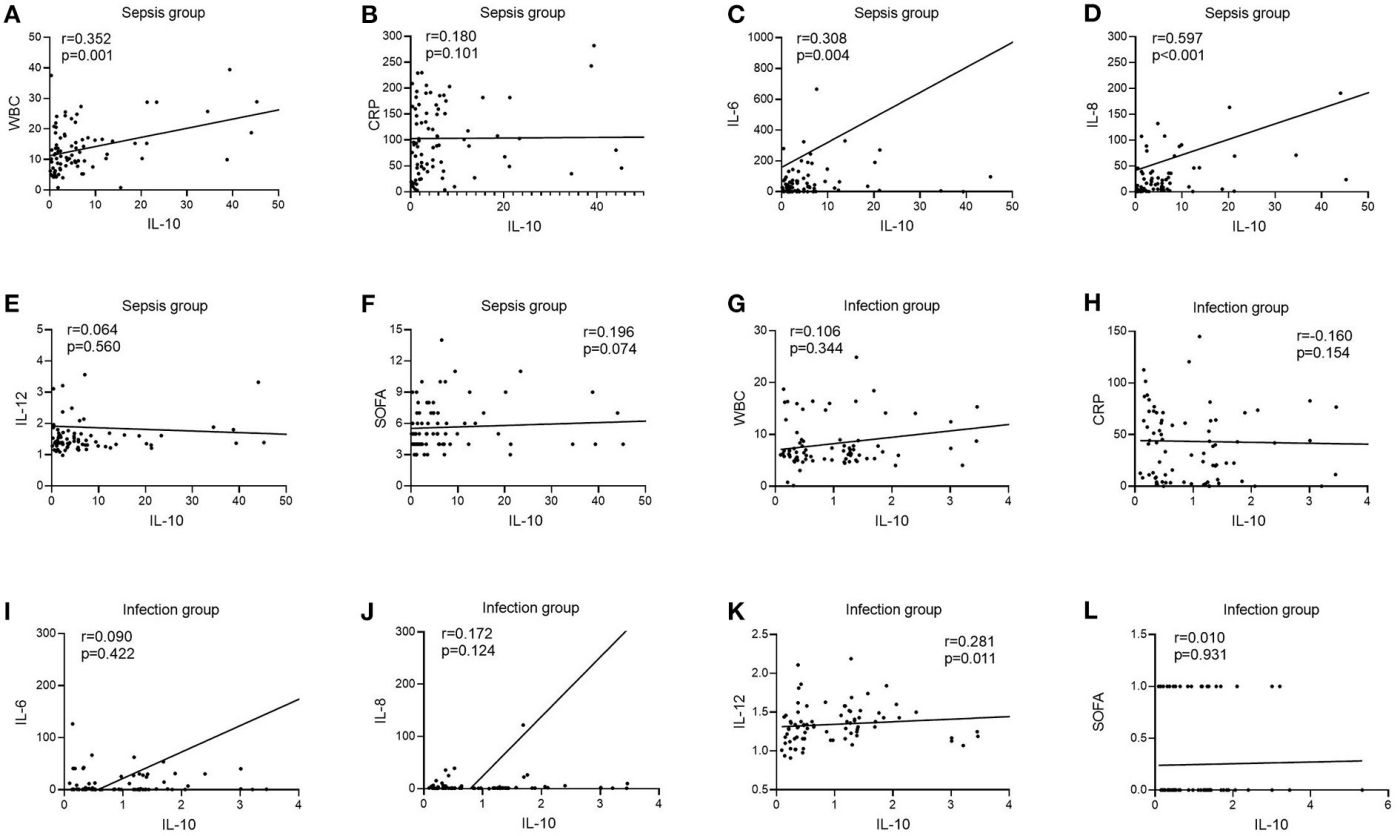
Correlation of IL-10 with WBC, CRP, IL-6, IL-8, IL-12, and SOFA scores in patients in the infection and sepsis groups. **(A)** Correlation of IL-10 with WBC in patients in the sepsis groups; **(B)** Correlation of IL-10 with CRP in patients in the sepsis groups; **(C)** Correlation of IL-10 with IL-6 in patients in the sepsis groups; **(D)** Correlation of IL-10 with IL-8 in patients in the sepsis groups; **(E)** Correlation of IL-10 with IL-12 in patients in the sepsis groups; **(F)** Correlation of IL-10 with SOFA scores in patients in the sepsis groups; **(G)** Correlation of IL-10 with WBC in patients in the infection groups; **(H)** Correlation of IL-10 with CRP in patients in the infection groups; **(I)** Correlation of IL-10 with IL-6 in patients in the infection groups; **(J)** Correlation of IL-10 with IL-8 in patients in the infection groups; **(K)** Correlation of IL-10 with IL-12 in patients in the infection groups; **(L)** Correlation of IL-10 with SOFA scores in patients in the infection groups.

There was no correlation between IL-10 levels and WBC, CRP, IL-6, IL-8, and SOFA scores in patients in the infection group [*r* = 0.106, *P* = 0.344 (Figure 3G); *r* = −0.160, *P* = 0.154 (Figure 3H); *r* = 0.090, *P* = 0.422 (Figure 3I); *r* = 0.172, *P* = 0.124 (Figure 3J); *r* = 0.010, *P* = 0.931 (Figure 3L)], and patients in the infection group IL-10 levels were positively correlated with IL-12 [*r* = 0.281, *P* = 0.011 (Figure 3K)]; patients in the sepsis group had no correlation between IL-10 levels and CRP, IL-12, and SOFA scores [*r* = 0.180, *P* = 0.101 (Figure 3B); *r* = 0.064, *P* = 0.560 (Figure 3E); *r* = 0.196, *P* = 0.074 (Figure 3F)], and patients in the sepsis group had no correlation between IL- 10 levels were positively correlated with WBC, IL-6, and IL-8 scores [*r* = 0.352, *P* = 0.001 (Figure 3A); *r* = 0.308, *P* = 0.004 (Figure 3C); *r* = 0.597, *P* < 0.001 (Figure 3D)].

### Correlation of IL-17 levels with other laboratory-related infection indicators in two groups of patients

**Figure 4:**
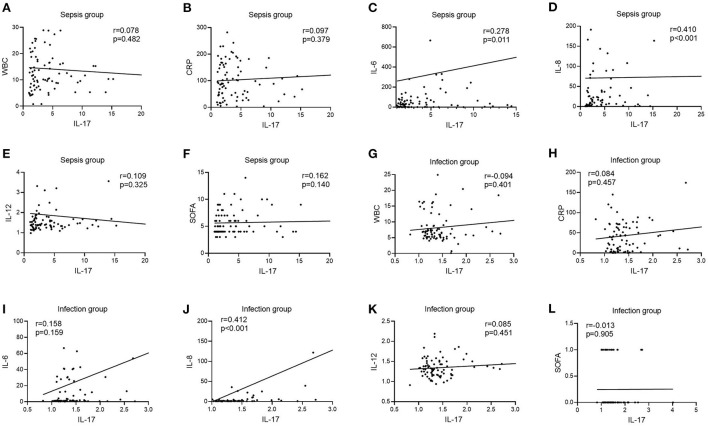
Correlation of IL-12 with WBC, CRP, IL-6, IL-8, IL-12, and SOFA scores in patients in the infection and sepsis groups. **(A)** Correlation of IL-12 with WBC in patients in the sepsis groups; **(B)** Correlation of IL-12 with CRP in patients in the sepsis groups; **(C)** Correlation of IL-12 with IL-6 in patients in the sepsis groups; **(D)** Correlation of IL-12 with IL-8 in patients in the sepsis groups; **(E)** Correlation of IL-12 with IL-12 in patients in the sepsis groups; **(F)** Correlation of IL-12 with SOFA scores in patients in the sepsis groups; **(G)** Correlation of IL-12 with WBC in patients in the infection groups; **(H)** Correlation of IL-12 with CRP in patients in the infection groups; **(I)** Correlation of IL-12 with IL-6 in patients in the infection groups; **(J)** Correlation of IL-12 with IL-8 in patients in the infection groups; **(K)** Correlation of IL-12 with IL-12 in patients in the infection groups; **(L)** Correlation of IL-12 with SOFA scores in patients in the infection groups.

In the infection group, there was no link between IL-17 levels and WBC, CRP, IL-6, IL-12, or SOFA scores [*r* = −0.094, *P* = 0.401 (Figure 4G); *r* = 0.084, *P* = 0.457 (Figure 4H); *r* = 0.158, *P* = 0.159 (Figure 4I); *r* = 0.085, *P* = 0.451 (Figure 4K); *r* = −0.013, *P* = 0.905 (Figure 4L)], however IL-17 levels were favorably connected with IL-8 (*r* = 0.412, *P* < 0.001) (Figure 4J). In the sepsis group, there was no link between IL-17 levels and WBC, CRP, IL-12, or SOFA scores [*r* = 0.078, *P* = 0.482 (Figure 4A); *r* = 0.097, *P* = 0.379 (Figure 4B); *r* = 0.109, *P* = 0.325 (Figure 4E); *r* = 0.162, *P* = 0.140 (Figure 4F)], however IL-17 levels were positively connected with IL-6 and IL-8 (*r* = 0.278, *P* = 0.278) [*r* = 0.278, *P* = 0.011 (Figure 4C); *r* = 0.410, *P* < 0.001 (Figure 4D)].

### Correlation of PCT levels with other laboratory-related infection indicators in two groups of patients

**Figure 5:**
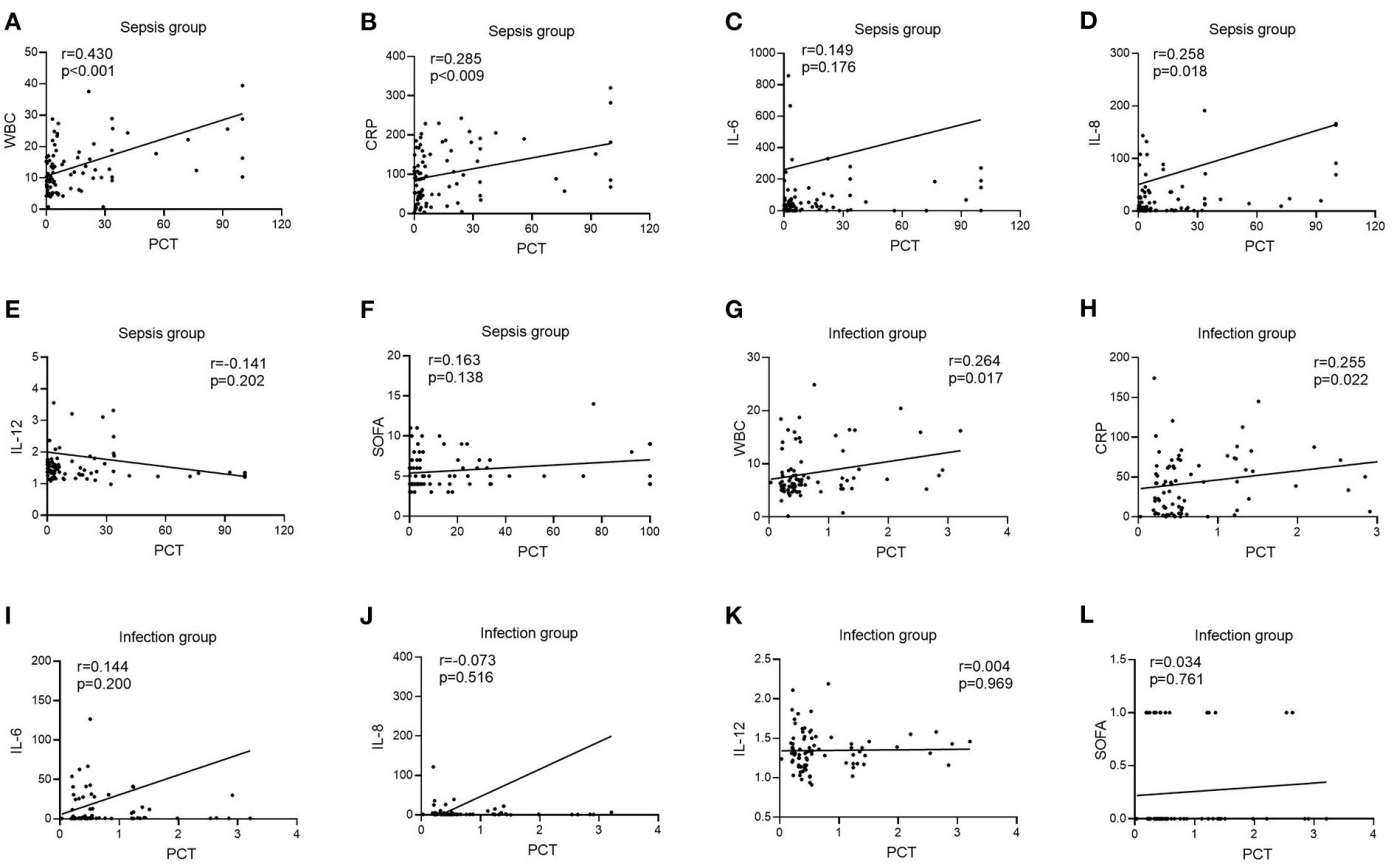
Correlation of PCT with WBC, CRP, IL-6, IL-8, IL-12, and SOFA scores in patients in the infection and sepsis groups. **(A)** Correlation of PCT with WBC in patients in the sepsis groups; **(B)** Correlation of PCT with CRP in patients in the sepsis groups; **(C)** Correlation of PCT with IL-6 in patients in the sepsis groups; **(D)** Correlation of PCT with IL-8 in patients in the sepsis groups; **(E)** Correlation of PCT with IL-12 in patients in the sepsis groups; **(F)** Correlation of PCT with SOFA scores in patients in the sepsis groups; **(G)** Correlation of PCT with WBC in patients in the infection groups; **(H)** Correlation of PCT with CRP in patients in the infection groups; **(I)** Correlation of PCT with IL-6 in patients in the infection groups; **(J)** Correlation of PCT with IL-8 in patients in the infection groups; **(K)** Correlation of PCT with IL-12 in patients in the infection groups; **(L)** Correlation of PCT with SOFA scores in patients in the infection groups.

In the infection group, there was no link between PCT levels and IL-6, IL-8, IL-12, or SOFA scores [*r* = 0.144, *P* = 0.200 (Figure 5I); *r* = −0.073, *P* = 0.516 (Figure 5J); *r* = 0.004, *P* = 0.969 (Figure 5K); *r* = 0.034, *P* = 0.761 (Figure 5L)], although there was a positive correlation between PCT levels and WBC and CRP [*r* = 0.264, *P* = 0.017 (Figure 5G); *r* = 0.255, *P* = 0.022 (Figure 5H)]. PCT levels were not connected with IL-6, IL-12, or SOFA ratings in the sepsis group [*r* = 0.149, *P* = 0.176 (Figure 5C); *r* =−0.141, *P* = 0.202 (Figure 5E); *r* = 0.163, *P* = 0.138 (Figure 5F)], but were positively correlated with WBC, CRP, and IL-8 [*r* = 0.430, *P* < 0.001 (Figure 5A); *r* = 0.285, *P* = 0.009 (Figure 5B); *r* = 0.258, *P* = 0.018 (Figure 5D)].

### Diagnostic utility of LASSO regression variables in individuals with sepsis vs. infection groups

The infection subgroup Y (sepsis group = 1, infection group = 0) was used as the response variable, and IL-10 (X1), IL-17 (X2), and PCT (X3) were used as independent variables. Binary logistic regression analysis was used to calculate the joint predictors of IL-10, IL-17, and PCT, and the regression equation was Y = −6.156 + 0.652X1 + 1.369X2 + 1.241X3, and the joint predictors were used as indicators of the three combined tests for outcome analysis.

**Figure 6:**
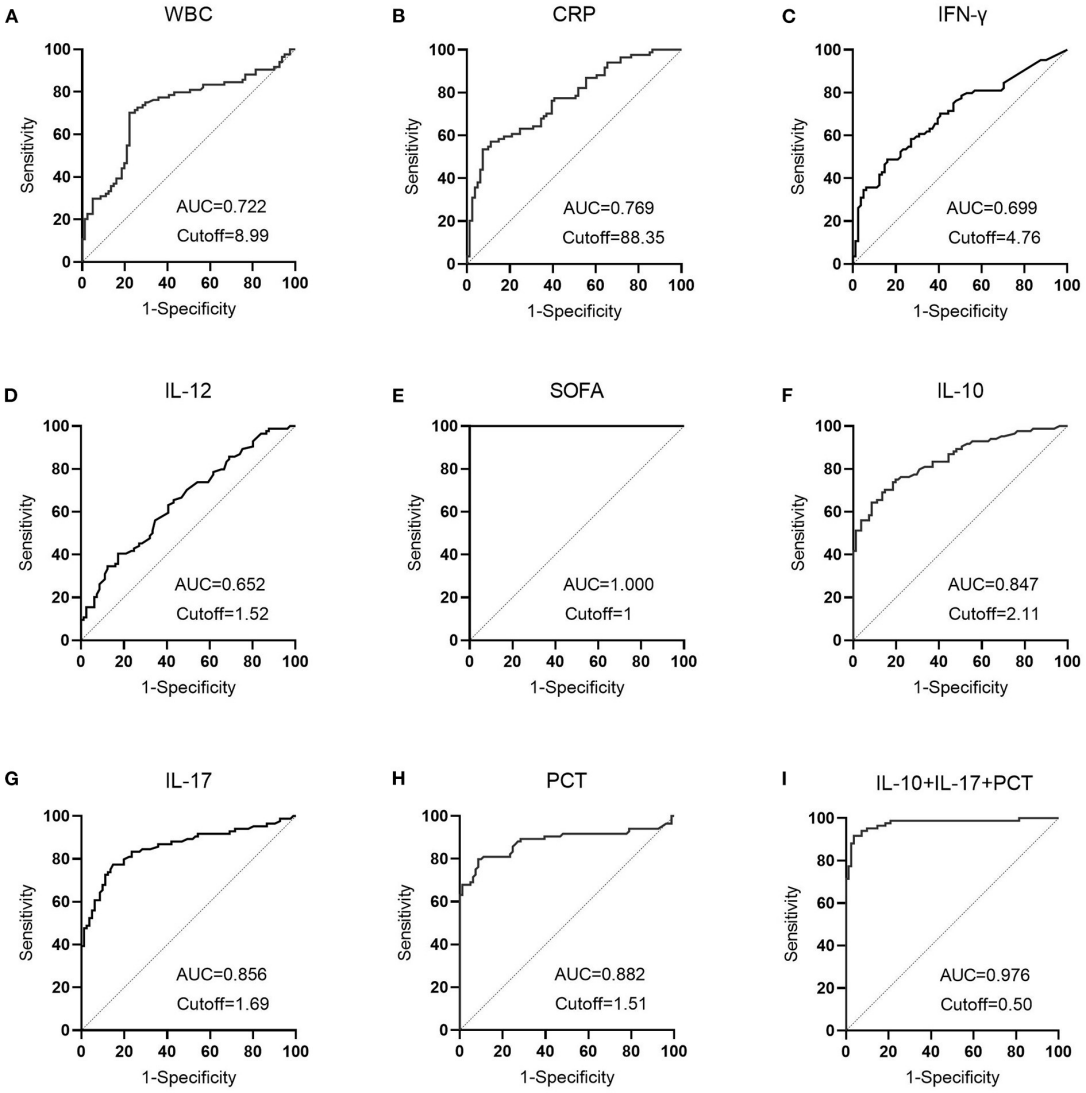
The diagnostic performance of LASSO regression analysis variables in patients with sepsis vs. infection. **(A)** The diagnostic performance of WBC in patients in the sepsis and infection group; **(B)** The diagnostic performance of CRP in patients in the sepsis and infection group; **(C)** The diagnostic performance of IFN-γ in patients in the sepsis and infection group; **(D)** The diagnostic performance of IL-12 in patients in the sepsis and infection group; **(E)** The diagnostic performance of SOFA in patients in the sepsis and infection group; **(F)** The diagnostic performance of IL-10 in patients in the sepsis and infection group; **(G)** The diagnostic performance of IL-17 in patients in the sepsis and infection group; **(H)** The diagnostic performance of PCT in patients in the sepsis and infection group; **(I)** The diagnostic performance of IL-10+IL-17+PCT in patients in the sepsis and infection group.

ROC curves were created by LASSO regression analysis of the seven variables tested (PCT, WBC, CRP, IL-10, IFN-γ, IL-12, IL-17), SOFA scores, and three combination (IL-10 + IL-17 + PCT) indicators (Figure 6). The sensitivity and specificity of the PCT assay with an AUC value of 0.882 (Figure 6H) and a cutoff value of 1.51 ng/mL were 79.76 and 91.36%, and the NPV and PPV were 81.3 and 90.5%, respectively. the sensitivity and specificity of the WBC assay with an AUC value of 0.722 (Figure 6A) and a cutoff value of 8.99 × 10^9^/L were The AUC value of CRP assay was 0.769 (Figure 6B) and the cutoff value was 88.35 mg/L, the sensitivity and specificity were 53.57 and 92.59%, and the NPV and PPV were 65.8 and 88.2%, respectively. The sensitivity and specificity were 64.29, 91.36, 71.2, and 88.5% for NPV and PPV, respectively, at an AUC value of 0.847 (Figure 6C) and a cutoff value of 2.11 pg/mL for IFN-γ assay. The AUC value of IL-12 assay was 0.652 (Figure 6D) and the cutoff value was 1.52 pg/mL, the sensitivity and specificity were 40.48 and 82.72%, and the NPV and PPV were 57.3 and 70.8%, respectively. The sensitivity and specificity were 77.38, 85.19, 78.4, and 84.4% for NPV and PPV, respectively, at an AUC value of 0.856 (Figure 6G) and a cutoff value of 1.69 pg/mL. The sensitivity and specificity were 100.00, 100.00, 100.00% for NPV and PPV, respectively, at an AUC value of 1.000 (Figure 6E) and a cutoff value of 1 for SOFA score. The AUC value of IL-10 + IL-17 + PCT was 0.976 and the cutoff value was 0.50 (Figure 6I), the sensitivity and specificity were 91.67 and 96.30%, and the NPV and PPV were 91.8 and 96.2%, respectively (Table 2).

**Table 2:** Comparative analysis of the results of each index in patients in the sepsis and infection groups.

**Items**	**Youden index**	**Cutoff value**	**AUC**	**Sensitivity (%)**	**Specificity (%**)	**AUC 95%CI**	**NPV (%)**	**PPV (%)**
PCT	0.711	1.51	0.882	79.76	91.36	0.882–0.927	81.3	90.5
WBC	0.480	8.99	0.722	70.24	77.78	0.647–0.789	71.6	76.6
CRP	0.769	88.35	0.769	53.57	92.59	0.697–0.831	65.8	88.2
IL-10	0.556	2.11	0.847	64.29	91.36	0.783–0.898	71.2	88.5
IFN-γ	0.328	4.76	0.699	48.81	83.95	0.623–0.768	61.3	75.9
IL-12	0.232	1.52	0.652	40.48	82.72	0.574–0.724	57.3	70.8
IL-17	0.626	1.69	0.856	77.38	85.19	0.749–0.906	78.4	84.4
SOFA	1.000	1	1.000	100.00	100.00	0.978–1.000	100.00	100.00
Combined assay	0.880	0.50	0.976	91.67	96.30	0.940–0.994	91.8	96.2

Table 2 demonstrates that the AUC values of IL-10, IL-17, PCT, and the combination test of IL-10 + IL-17 + PCT were higher and had superior diagnostic performance. This finding is consistent with LASSO-logistic regression screening PCT, IL-10, and IL-17 as separate risk factors for the development of sepsis. The AUCs of IL-10, IL-17, PCT, and IL-10 + IL-17 + PCT combination tests were analyzed using Medcalc software, and there was no statistically significant difference in the AUCs of IL-10, IL-17, and PCT assays (*P* > 0.05). The AUCs of the IL-10+IL-17+PCT combination were bigger than the AUCs of the IL-10, IL-17, and PCT assays alone, and the difference was statistically significant (*P* < 0.05) (Table 3).

**Table 3:** Comparison of the AUC areas of IL-10, IL-17, PCT and IL-10+IL-17+PCT combined assays.

**Items**	***Z-*** **value**	***P-*** **value**
Combined assay and IL-10	4.392	<0.001
Combined assay and IL-17	4.162	<0.001
Combined assay and PCT	3.460	<0.001
IL-10 and IL-17	0.272	0.786
IL-10 and PCT	0.797	0.425
IL-17 and PCT	0.568	0.570

### The clinical decision curve analysis of IL-10, IL-17, PCT and three combined tests in the development of sepsis in infected patients

**Figure 7:**
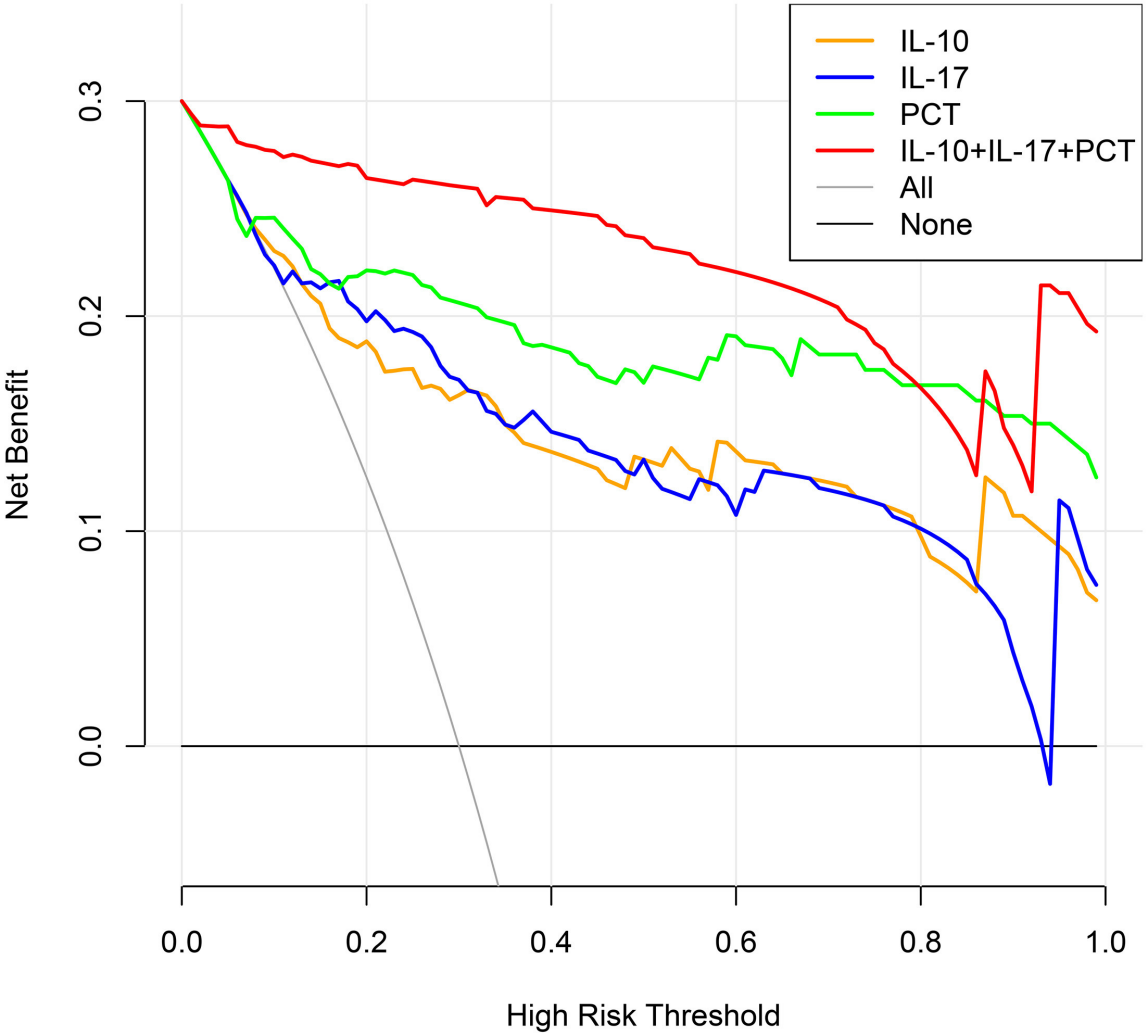
Clinical decision-making curves for the development of sepsis in infected individuals were analyzed (X: probability with value, Y: net benefit, Black line: hypothesis of no sepsis in all patients, Gray line: hypothesis of sepsis in all patients).

The clinical decision curve for sepsis in infected patients demonstrated that when the threshold probability of IL-10 detection alone was 8%-100%, the threshold probability of IL-17 detection alone was 6%-93 %, the threshold probability of PCT detection alone was 6%-100 %, and the probability of the three combined detection thresholds was 1%-100 %, the predictive model's net clinical benefit was significant. These findings imply that the aggregate clinical benefit of the three studies is greater than the benefit of the single trial (Figure 7).

### Columnar plots for sepsis risk prediction using IL-10, IL-17, and PCT

**Figure 8:**
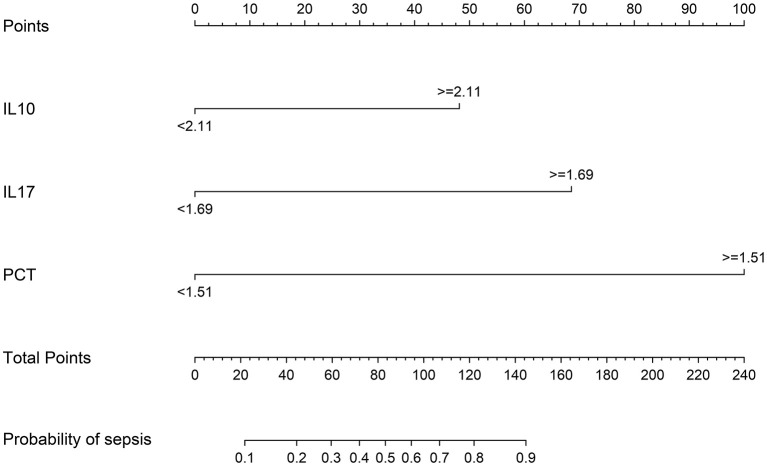
The nomogram predicting the risk of sepsis in infected patients.

The meaningful variables IL-10, IL-17, and PCT were screened out from the multifactorial analysis and included in the prediction model. Since IL-10, IL-17, and PCT were non-normal data, the cutoff values calculated from the ROC curves were used as the cutoff points of the variables, and the column line plots were constructed after dichotomous transformation (Figure 8). The scores corresponding to the column line plots were 48 for IL-10 ≥ 2.11, 69 for IL-17 ≥ 1.69, and 100 for IL-17 ≥ 1.51, respectively, and the total scores of 22, 44, 60, 72, 83, 95, 107, 122, and 145 corresponded to a probability of sepsis of 10–90%, respectively.

### The validation of a predictive model for the risk of sepsis in infected patients

**Figure 9:**
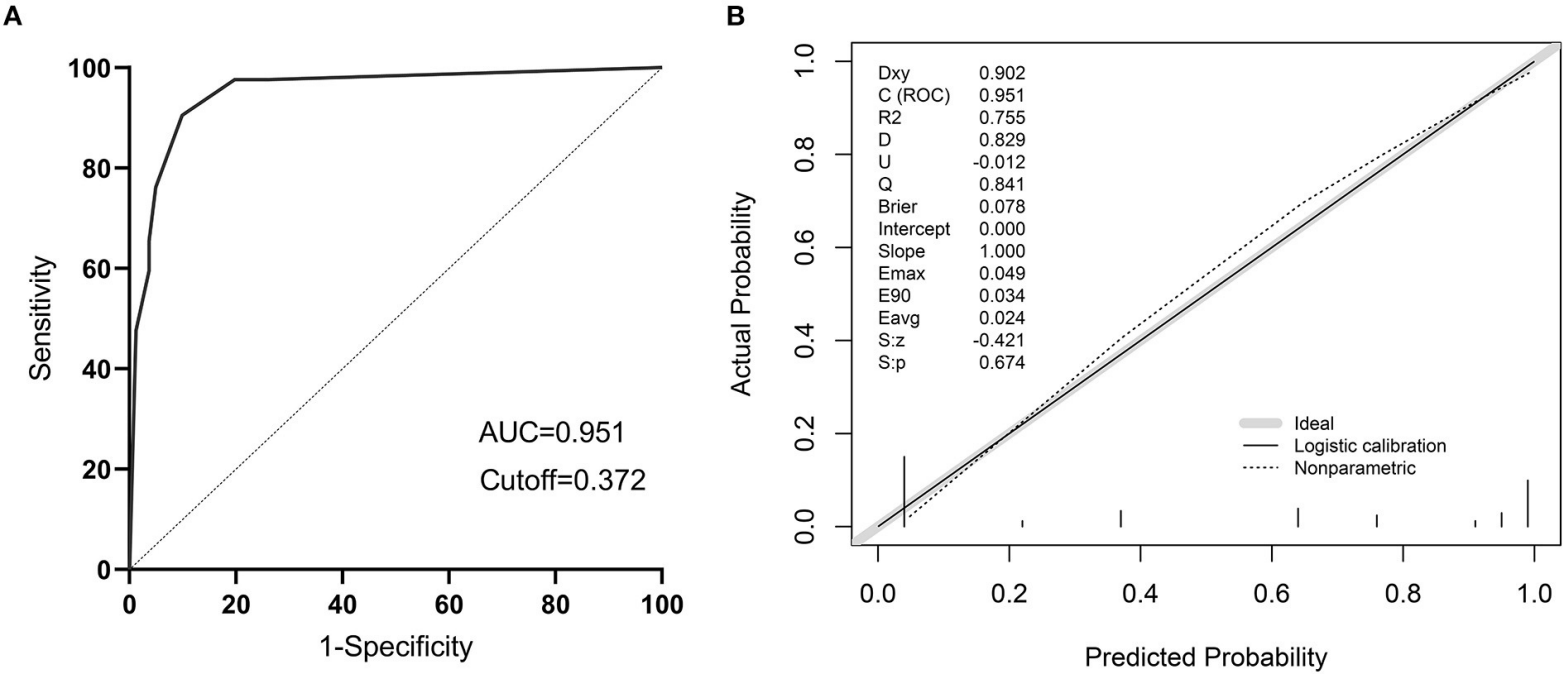
The validation of a predictive model for the risk of sepsis in infected patients. **(A)** ROC curve of the prediction model for the risk of sepsis in infected patients; **(B)** Calibration curve of the model for the risk of sepsis in infected patients.

The models were evaluated using discrimination and calibration. The discrimination was evaluated by calculating the area under the subject operating characteristic (ROC) curve (AUC) results for the predicted probabilities. The AUC value of the model was 0.951 (0.906–0.979), indicating that the prediction model has good discrimination (Figure 9A). The calibration degree of the prediction model is the agreement of the predicted probability with the actual observation. The calibration degree showed the consistency of the prediction model's risk predicted values with the actual observed values through the Calibration calibration curve and calculation of C-index, Hosmer-Lemeshow test results. The Hosmer-Lemeshow test results showed no statistically significant difference between the model's risk predicted values and the actual observed values (χ^2^= 5.936, *P* = 0.746), with a C-index value of 0.951 (95% CL: 0.919–0.983). Through internal validation of the model data, the Calibration calibration curve showed that the predicted probabilities of the model were in good agreement with the actual probabilities (Figure 9B).

### Clinical utility of a model for risk of sepsis in infected patients

**Figure 10:**
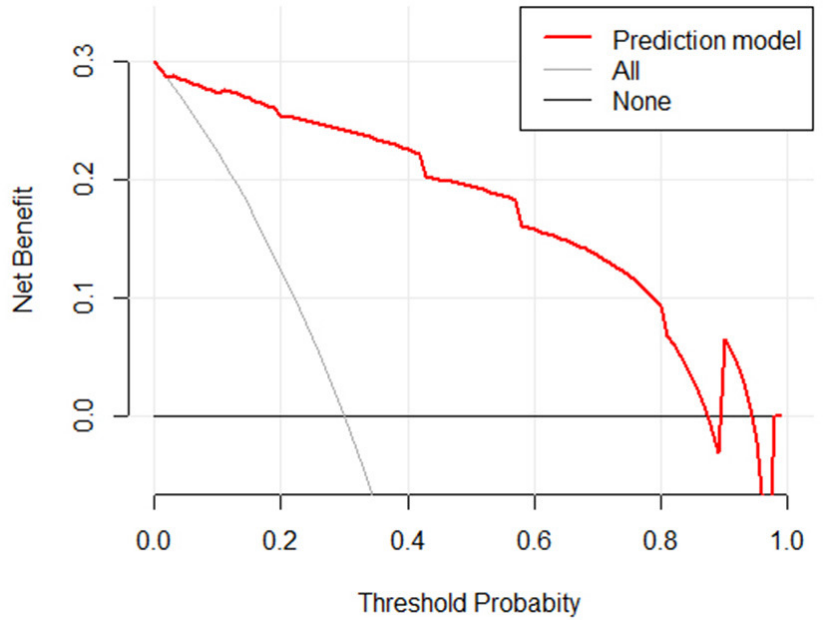
Clinical decision curve for the risk of sepsis in infected patients. (X: probability with value, Y: net benefit, black line: hypothesis of no sepsis in all patients, gray line: hypothesis of sepsis in all patients).

The clinical decision curves for the risk of sepsis in infected patients model showed that when the model threshold probabilities were 3–87%, the net clinical benefit of the prediction model was greater in all cases than in all patients with sepsis or all without sepsis scenarios (Figure 10).

## Discussion

There are several concerns with sepsis, an infection-induced disease that causes organ malfunction and may lead to death in critical care units, and the medical profession is grappling with them all (20). Due to the fact that clinical symptoms are sometimes inadequately exhibited or delayed in their emergence, the optimal period for therapy is frequently missed at the time of diagnosis; yet, early detection enables early pharmacological intervention and improves patient survival. According to reports, the death rate of patients rises by about 7.6% for every 1 h delay in antibiotic therapy during the first 6 h following the beginning of sepsis, and the efficacy of antibiotic treatment is not sufficient beyond 24 h after the onset of sepsis (21). The irrational use of antibiotics may result in dangerous side effects, and the long-term irrational use of antibiotics can result in a rise in antibiotic resistance. As a result, early identification and diagnosis of sepsis is very important in the clinical management of this condition (22).

The 17 clinical and infection-related variables, including age and gender, were analyzed in this research. An independent risk of sepsis development was shown to be associated with the presence of three markers: interleukin 10, interleukin 17, and procalcitonin (PCT). IL-10 has been proposed as a possible diagnostic marker in the early stages of infection and has been shown to increase the sensitivity and specificity of early sepsis diagnosis (23). By increasing neutrophil movement and other methods, IL-17 may play a significant role in the immune response (24). Patients in critical care units often utilize PCT as an indication of infection and antibiotic treatment (25). IL-10, PCT, and PCT have been shown to have an essential role in infectious disorders, which is consistent with this study's findings.

As a result of the study, the infection group had higher IL-10 levels than the sepsis group; this correlation was shown to be more pronounced in the sepsis group than it was in the infection group. WBC, IL-6, and IL-8 were regularly done in most main units, demonstrating that IL-10 has a good value in sepsis infections. IL-10 was also related with greater inflammatory markers in the sepsis group. According to the research, IL-10 is produced by a variety of cells and has a crucial function in limiting the production of pro-inflammatory cytokines, such as IL-12, which are generated when a person is inflamed (26). In the infected group, there was a positive correlation between IL-17 levels and IL-8, but in the sepsis group, there was a positive correlation between IL-6 and IL-8, showing that IL-17 is better able to react to inflammation markers. This cytokine is both protective and harmful, stimulating neutrophils and macrophages at the site of infection to achieve anti-infective effects. An key part of the body's defense against different pathogens (27). Predictors of infectious illness, disease severity, and antimicrobial medication usage were all positively connected with PCT levels in the infected group, and PCT is an essential diagnostic marker in the diagnosis of infectious diseases, disease severity, and antimicrobial drug use (28, 29). Infectious indices, correlation indicates their usefulness.

The susceptibility and severity of sepsis are associated with cytokines that correlate with most signs and symptoms of the systemic inflammatory response in patients with sepsis, so differences in cytokine expression in response to infection may be a factor in the variability of patients' clinical features (30). IL-10 is an anti-inflammatory cytokine that plays a key role in the pathogenesis of sepsis. Normally, IL−10 inhibits the activation and expansion of T cells and acts as a suppressor of factors released by activated monocytes, such as pro-inflammatory cytokines, chemokines, and polymorphonuclear leukocytes and eosinophils released during the inflammatory response (31). The effects of IL−10 on the immune response include downregulation of key signaling receptors on antigen-presenting cells such as CD40, CD80, CD86, and MHC II, reduction of Mac-1 expression and inhibition of neutrophil oxidative burst, inhibition of T cell proliferation and IL−2, IL−6, and IFN-γ production, maintenance of FoxP3 expression in regulatory T cells, and inhibition of NK cell function (32). Thus, IL-10 may counteract the negative effects of excessive cytokine production in sepsis patients and play an important role in the pathogenesis of sepsis. IL-17 is a pro-inflammatory cytokine originally identified as a T cell-derived molecule to activate IL-6 production. Secretion of IL-17 is found in a variety of cell types, including T helper cells (Th17), NK cells, γδ-T cells, mast cells, and Th2 cells. Clinical studies have linked IL-17 to the pathogenesis of inflammatory diseases (33). Sepsis is characterized by immune system dysfunction associated with systemic cytokine levels and lymphocyte apoptosis, with pro- and anti-inflammatory cytokines released early in sepsis and synchronized with disease progression throughout its course (34). Pro-inflammatory cytokines play an important role in the pathogenesis of sepsis. These endogenous mediators prolong the inflammatory response and their overproduction can lead to death. Anti-inflammatory cytokines are produced to protect our body from severe damage caused by pro-inflammatory cytokines. When they are produced in increased amounts, the host becomes immunosuppressed, leading to death. When monitoring the progression of the inflammatory response, the concentrations of both pro- and anti-inflammatory cytokines must be determined (35).

The 7 variables (PCT, WBC, CRP, IL-10, IFN-, IL-12, IL-17), SOFA scores, and three combination indicators (IL-10+IL-17+PCT) were screened using LASSO regression analysis to yield ROC curves (response area curves). These three indices were shown to be superior to other laboratory indices in the diagnosis of early sepsis, with AUC values of IL-10, IL-17 and PCT and three combined tests over 80% and negative predictive values above 70%, respectively. A two-by-two comparison of the AUCs of IL-10, IL-17, PCT, and the three combined tests revealed no statistically significant difference (*P* > 0.05), suggesting that the three indications were virtually the same in terms of diagnostic ability. The AUC of the combination of the three tests, IL-10 + IL-17 + PCT, was larger than the AUC of IL-10, IL-17, and PCT alone (*P* < 0.05). Furthermore, the negative predictive values were all >90%, indicating that the three combined tests provide optimum diagnostic performance. As a final step, clinical decision curve analysis was done for the three combined tests, and the three combined tests were shown to have a higher overall clinical benefit rate than single tests, indicating that combined tests are more effective than single tests.

On the basis of IL-10, IL-17, and PCT, a nomogram for predicting the risk of sepsis incidence is constructed. Due to the non-biased distribution of IL-10, IL-17, and PCT, the cutoff value determined from the ROC curve is utilized as the cutoff point for the variables, and the nomogram is generated following dichotomous transformation. By simply adding to the graph, one may analyze the chance of developing sepsis in a straightforward and clear manner. The scores for the three factors are added to generate a total score, and the total score is used to draw a vertical line toward the probability line to determine the chance of sepsis happening in the patient. The nomogram model's AUC score was 0.951, suggesting that the prediction model has a high degree of discriminating. In terms of calibration accuracy, the projected probabilities of the model were in excellent agreement with the actual probabilities. Clinical utility investigation of this nomogram model revealed that it provided a significant net clinical benefit when the threshold probability was between 3 and 87%.

In summary, there is excellent diagnostic value in patients with sepsis for IL-10, IL-17, and PCT testing; the combination of these three tests is better than the individual tests in terms of diagnostic performance, while the total clinical benefit rate of the three tests is better than the individual tests. First and foremost, this study is a retrospective study with a small sample size, and there is a possibility of bias in diagnostic value as well as unavoidable selection bias, and further sample collection and prospective design studies are needed to better evaluate how each index contributes to the diagnosis of sepsis. We still need to expand the sample size and assess and verify the model at additional medical institutions in the future, even though we have internally tested the model with excellent discrimination and calibration.

## Data availability statement

The raw data supporting the conclusions of this article will be made available by the authors, without undue reservation.

## Ethics statement

The study was approved by the Ethics Committee of Shanghai Tongji Hospital (2021KYSB061) and the methods were designed according to the Declaration of Helsinki. Written informed consent to participate in this study was provided by the participants' legal guardian/next of kin. The patients/participants provided their written informed consent to participate in this study.

## Author contributions

WZ: formal analysis, validation, investigation, visualization, methodology, and writing-original draft and editing. WH and WW: data curation and methodology. CJ and JH: methodology. LS: investigation and supervision. LH: data curation and resources. JW and AS: conceptualization, resources, supervision, funding acquisition, project administration, and writing-review and editing.

## Funding

This work was supported by the Postdoctoral Science Foundation of China (2020M681399), the Zhejiang Medicine and Health Project (2019KY164), and LS Master Teacher's Studio.

## Conflict of interest

The authors declare that the research was conducted in the absence of any commercial or financial relationships that could be construed as a potential conflict of interest.

## Publisher's note

All claims expressed in this article are solely those of the authors and do not necessarily represent those of their affiliated organizations, or those of the publisher, the editors and the reviewers. Any product that may be evaluated in this article, or claim that may be made by its manufacturer, is not guaranteed or endorsed by the publisher.

## Supplementary material

Supplementary Figure S1 Screening flow chart of patients in infection group and sepsis group.
