# Supplementary figures and images for: The diagnostic utility of IL-10, IL-17, and PCT in patients with sepsis infection
Source: Front Public Health. 2022 Jul 22;10:923457. doi: 10.3389/fpubh.2022.923457 (PMC9355284; doi:10.3389/fpubh.2022.923457)

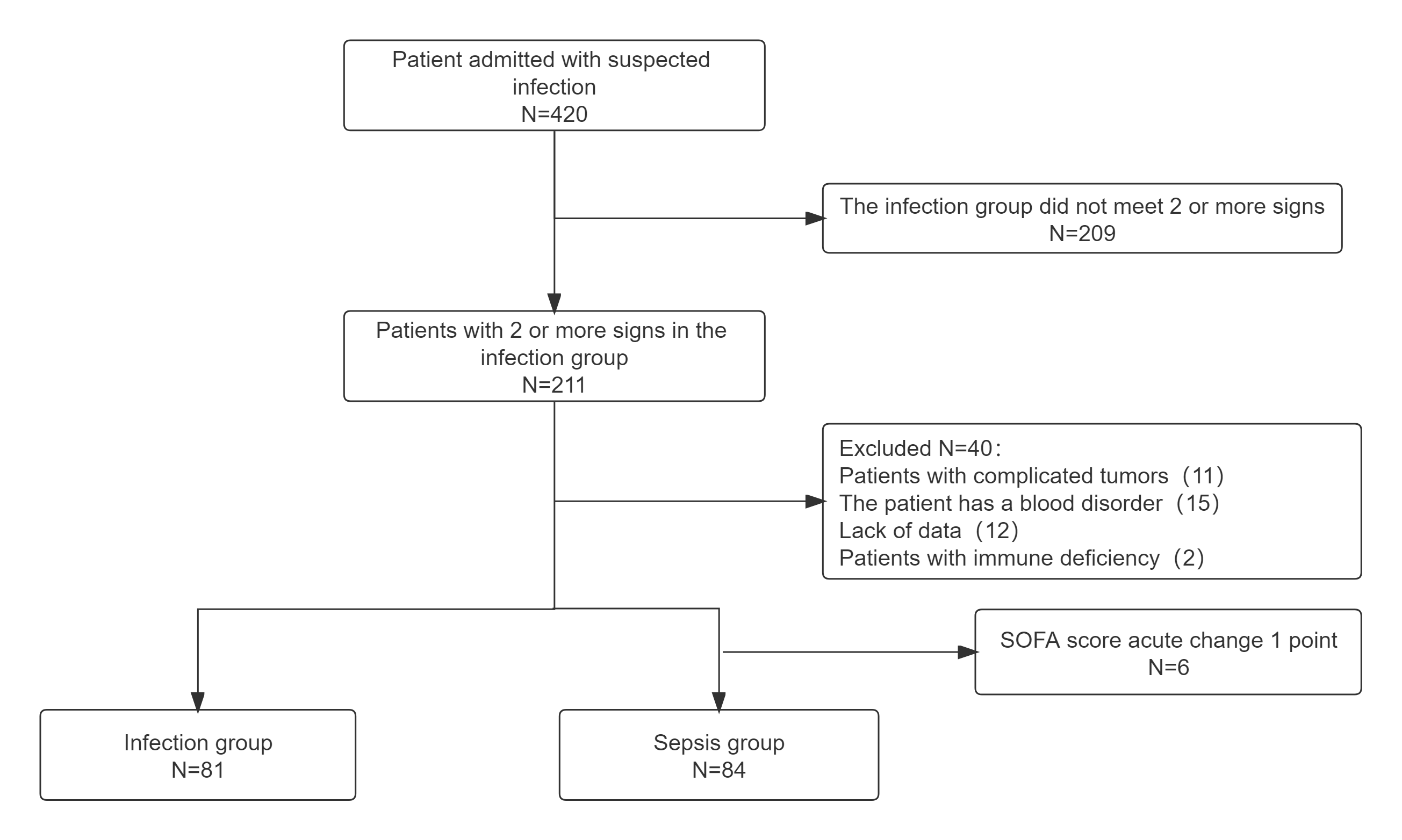

Supplement: Supplementary Figure S1 — Screening flow chart of patients in infection group and sepsis group. [file Image_1.PNG]
